# Using Location Intelligence to Evaluate the COVID-19 Vaccination Campaign in the United States: Spatiotemporal Big Data Analysis

**DOI:** 10.2196/39166

**Published:** 2023-02-16

**Authors:** Qingfeng Li, James Cheng Peng, Diwakar Mohan, Brennan Lake, Alex Ruiz Euler, Brian Weir, Lena Kan, Cui Yang, Alain Labrique

**Affiliations:** 1 Department of International Health Johns Hopkins Bloomberg School of Public Health Baltimore, MD United States; 2 Department of Applied Mathematics and Statistics Johns Hopkins Whiting School of Engineering Baltimore, MD United States; 3 Cuebiq – Intelligence in Action New York, NY United States; 4 Department of Health, Behavior and Society Johns Hopkins Bloomberg School of Public Health Baltimore, MD United States

**Keywords:** COVID-19 vaccine, vaccine campaign, big data, vaccination, vaccine, COVID-19, uptake, effectiveness, barriers, hesitancy, health information

## Abstract

**Background:**

Highly effective COVID-19 vaccines are available and free of charge in the United States. With adequate coverage, their use may help return life back to normal and reduce COVID-19–related hospitalization and death. Many barriers to widespread inoculation have prevented herd immunity, including vaccine hesitancy, lack of vaccine knowledge, and misinformation. The Ad Council and COVID Collaborative have been conducting one of the largest nationwide targeted campaigns (“It’s Up to You”) to communicate vaccine information and encourage timely vaccination across the United States. More than 300 major brands, digital and print media companies, and community-based organizations support the campaigns to reach distinct audiences.

**Objective:**

The goal of this study was to use aggregated mobility data to assess the effectiveness of the campaign on COVID-19 vaccine uptake.

**Methods:**

Campaign exposure data were collected from the Cuebiq advertising impact measurement platform consisting of about 17 million opted-in and deidentified mobile devices across the country. A Bayesian spatiotemporal hierarchical model was developed to assess campaign effectiveness through estimating the association between county-level campaign exposure and vaccination rates reported by the Centers for Disease Control and Prevention. To minimize potential bias in exposure to the campaign, the model included several control variables (eg, age, race or ethnicity, income, and political affiliation). We also incorporated conditional autoregressive residual models to account for apparent spatiotemporal autocorrelation.

**Results:**

The data set covers a panel of 3104 counties from 48 states and the District of Columbia during a period of 22 weeks (March 29 to August 29, 2021). Officially launched in February 2021, the campaign reached about 3% of the anonymous devices on the Cuebiq platform by the end of March, which was the start of the study period. That exposure rate gradually declined to slightly above 1% in August 2021, effectively ending the study period. Results from the Bayesian hierarchical model indicate a statistically significant positive association between campaign exposure and vaccine uptake at the county level. A campaign that reaches everyone would boost the vaccination rate by 2.2% (95% uncertainty interval: 2.0%-2.4%) on a weekly basis, compared to the baseline case of no campaign.

**Conclusions:**

The “It’s Up to You” campaign is effective in promoting COVID-19 vaccine uptake, suggesting that a nationwide targeted mass media campaign with multisectoral collaborations could be an impactful health communication strategy to improve progress against this and future pandemics. Methodologically, the results also show that location intelligence and mobile phone–based monitoring platforms can be effective in measuring impact of large-scale digital campaigns in near real time.

## Introduction

The COVID-19 pandemic has impacted every country in the world, and the United States was hit particularly hard. As of February 2022, more than 900,000 people had died in the United States after contracting the virus [1]. Several highly effective vaccines have been developed and authorized for emergency use by Food and Drug Administration [2,3]. In sharp contrast to the early days of the pandemic in the United States, there are fewer structural barriers that could interfere with vaccine coverage. However, attitudinal barriers remain prevalent [4]. According to a national poll conducted by the COVID Collaborative, although nearly 90% of Americans recognize the effectiveness of vaccines, only about a third plan to get vaccinated themselves [5]; and other surveys revealed similar patterns [6,7]. This mistrust is more prevalent among racial and ethnic minority communities. A nationwide poll found that only 14% of Black Americans and 34% of Latinx Americans trust the safety of the vaccines [8]. The massive distrust among racial and ethnic minority communities is the result, according to various analyses, of generational trauma and continued mixed messages about the pandemic and vaccines [9]. Latinx and African American communities have been disproportionately affected by the COVID-19 pandemic [10-12]. This needs to be addressed immediately since enhancing access and uptake of the COVID-19 vaccine among those most at risk is critical to control the epidemic, reduce the emergence of new variants, and promote health equity.

Similar hesitancy has been documented with nearly all vaccines [9,10]. Vaccine hesitancy is a multifactorial phenomenon, and therefore, requires multifaceted strategies. An effective and comprehensive campaign should be part of a national vaccination plan. Many states began dealing with vaccine hesitancy early in vaccine rollout, particularly in selected areas and among certain racial and ethnic groups. Information campaigns were developed to provide science-based information and culturally appropriate resources, either through existing medical networks or communication firms [11]. Some states also built or used partnerships with faith-based leaders and trusted community organizations to reach critical populations and increase vaccine acceptance among minority communities [11]. There are also other forms of media campaigns, primarily focusing on certain groups or areas [12].

At the national level, the Ad Council and the COVID Collaborative launched a nationwide vaccine education campaign called “It’s Up to You” in February 2021 [13]. As one of the largest public health efforts in US history, the campaign aims to educate the American public and build confidence in vaccines by communicating vaccine messages informed by the best science. In coordination with over 300 major brands, digital and print media companies, community-based organizations, medical experts, the Ad Council has been rolling out public service announcements across airwaves, publications, and social media. The public service announcements provide facts and correct information, answer questions, and preempt false narratives through platforms that the target groups regularly visit. To bridge the racial and ethnic gap in vaccine knowledge, the campaign particularly targets racial and ethnic minority communities with contents customized for distinct cultural and historical background. Campaign materials are available in 7 languages (English, Spanish, Simplified Chinese, Korean, Russian, Haitian Creole, and Vietnamese) on the campaign website. Interested readers are referred to Ad Council and the campaign website for more information about the campaign design and delivery.

As of September 19, 2021, the campaign received US $168 million in media support and related publicity and achieved 80 million engagements with messages via social and search [13]. Historically, the use of media campaigns to communicate public health knowledge and promote positive behaviors has shown varying degrees of effectiveness [14-16]. Without a systematic assessment, implementers lose the opportunity to identify potential gaps in campaign design and make strategic changes to message content and dissemination strategy. Therefore, our study aims to evaluate the effectiveness of this national campaign in promoting vaccine uptake, which could be used to inform the design and evaluation of other large-scale behavior change interventions.

## Methods

### Exposure Data

Monitoring the exposure to mass media campaigns is challenging due to the dynamic nature of the campaign rollout and massive coverage. Traditional monitoring approaches include surveys, interviews, and cohort studies [17,18]. However, the relatively slow turnaround makes the approaches more suitable for a retrospective evaluation but less able to generate timely and actionable feedback.

Emerging geospatial technologies present an opportunity for frequent and rapid data collection. We collect aggregated exposure data through Cuebiq’s privacy-preserving geospatial data and analytics platform. Cuebiq partners with over 100 location-centric smartphone apps, providing a path for users to opt in and provide informed consent for their anonymized and aggregated data to be used for research purposes. In addition to its General Data Protection Regulation and California Consumer Privacy Act–compliant data collection practices, Cuebiq applies additional privacy protections beyond simple anonymization to prevent the reidentification of individual users.

To measure campaign exposure, a digital breadcrumb known as a pixel is attached to creative media assets served through multiple advertisement media, including web, mobile, and in-app browsing. Cuebiq then generates impressions data and matches those impressions with its own panel of users in a privacy-preserving manner. Measurement data are accessed by researchers via the Cuebiq Workbench platform, an auditable sandbox environment that allows access for the querying of data and generation of aggregate, privacy-preserving outputs. The sandbox enables the creation of aggregate data at county levels, without the ability or need to create individual-level outputs.

The Cuebiq platform collects exposure information in a nearly continuous manner for each individual device. The exposure is then summarized by a binary indicator for each device, denoting whether or not it is exposed to the campaign. Finally, for each county, we calculate the number of exposed devices, the number of unexposed devices, and the exposure rate (ie, the proportion of exposed devices among all monitored devices) on a weekly basis. As described above, the “It’s Up to You” campaign uses both digital and print media to deliver the materials. Our exposure data only measure the exposure to digital contents.

The final analytical data set covers 3104 counties in 48 states and the District of Columbia. Hawaii and Alaska are excluded because the spatial model requires counties to have geographically adjacent neighbors. Ad Council campaigns and Cuebiq’s measurement campaign were launched in late February 2021, but the campaign did not reach national coverage until about one month later. Campaign activities and related exposures wound down rapidly after August. To avoid potential selection bias in early campaign rollout, our analyses only used data between March 29 and August 29, 2021. The panel’s width and length are adequate for us to include complex model components, such as the spatiotemporal correlation structure.

### Outcome Data

The outcome measure, vaccination rate, comes from Centers for Disease Control and Prevention’s COVID Data Tracker, which provides county-level vaccination rates on a daily basis. To remain consistent with the exposure measures described above, weekly averages are taken at the county level.

### Other Control Variables

Several socioeconomic and demographic indicators have been shown to influence vaccine uptake. This study explored the following: age (percentage of people 85 years or older); race and ethnicity (percentage of non-Hispanic White population); political affiliation (percentage of people who voted for democrats in the 2020 presidential election); income (median household income). The inclusion of covariates is based on literature review, data availability, and model diagnoses.

### Statistical Model

Bayesian hierarchical mixed-effects models are used to estimate the correlation between exposure and outcome measures at the county level. As an important feature of infectious diseases, county-level pandemic measures exhibit spatial autocorrelation [19]. Nearer counties are more similar than distant counties in terms of COVID-19 pandemic and vaccination. We will account for the spatial autocorrelation in the regression. However, the residual may still display spatial autocorrelation, which violates a key regression modeling assumption. Similarly, panel data typically display temporal autocorrelation. Therefore, our model includes a conditional autoregressive (CAR) component to account for the spatiotemporal autocorrection in the data. Essentially, the CAR component supplements the main effects model with a set of spatiotemporally autocorrelated random effects [20].

Our final model can be formally expressed as follows:




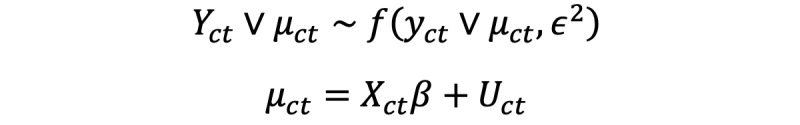




Here, where *c*=1,2,…, *c* indexes counties; *Y_ct_* denotes the vaccination rate (weekly and not cumulative) in week *t* in county *c*; *X_ct_* denotes regressors in week *t* in county *c*. Of note, some variables may be time invariant; *β* denotes a column vector of regression slopes.

A key model component is *ψ_ct_*, which induces the spatiotemporal autocorrelation for county-level measures after accounting for the covariate effects. In particular, we use a first-order autoregressive process where correlation is *ρ^w^*, *ρ*∈ (0,1) for *U*s that are *w* time units apart. Of particular interest is the spatial model that allows the *U_ct_* to be correlated with correlation depending on a distance metric. As for the first-order autoregressive time-series model, spatial models induce correlation among the *U_ct_* for a fixed *t.*

County coordinates are used to compute Euclidean distances. With relatively few nearby counties, a parameter model will estimate nearby correlations based on correlations between distant counties (an extrapolation). There are several model forms, including (powered) exponential, Gaussian, Spherical, Matern, and CAR models [21]. Due to the large number of contiguous counties in our data set, the CAR model worked well and was included in our analyses.

The model was fitted in R using the CARBayesST package [22]. The final results are extracted from 10,000 samples after discarding 10,000 burn-ins and thinning by 10 to reduce potential sample autocorrelation.

### Ethical Considerations

Study data are anonymous and deidentified, and the study did not constitute human subjects. Therefore, ethical approval was not required for this study.

## Results

**Figure 1:**
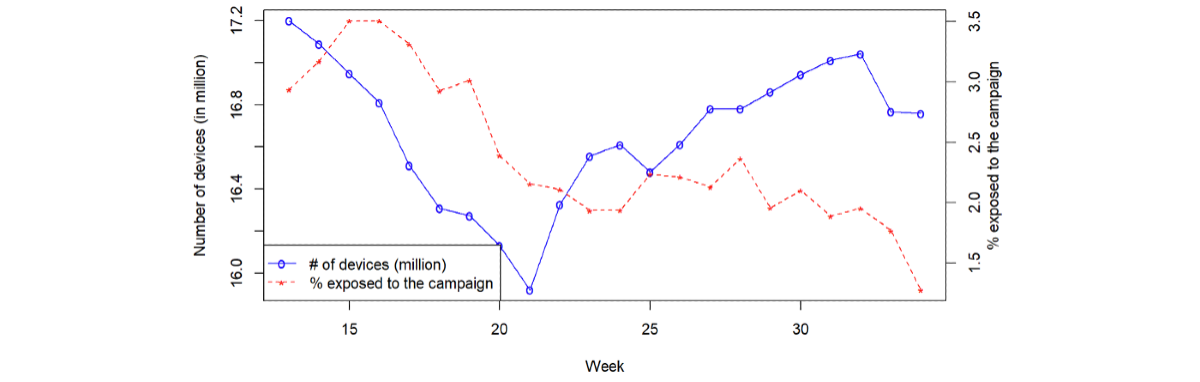
Number of opted-in, deidentified devices (in million) monitored by the Cuebiq platform and the percentage exposed to the Ad Council Campaign.

The campaign was launched in February 2021, starting with a small number of counties in the first few weeks. It reached national coverage in week 13 (March 29 to April 4, 2021), when about 99% (3058/3104) of counties in the Cuebiq platform detected exposure to the campaign. Campaign activities started winding down quickly from week 34 (August 23-29, 2021). As a result, this study covered a period of 22 weeks. Cuebiq continued monitoring a panel of about 17 million mobile devices (Figure 1). The panel remained stable during this period, ensuring the temporal comparability of the observed metrics.

**Figure 2:**
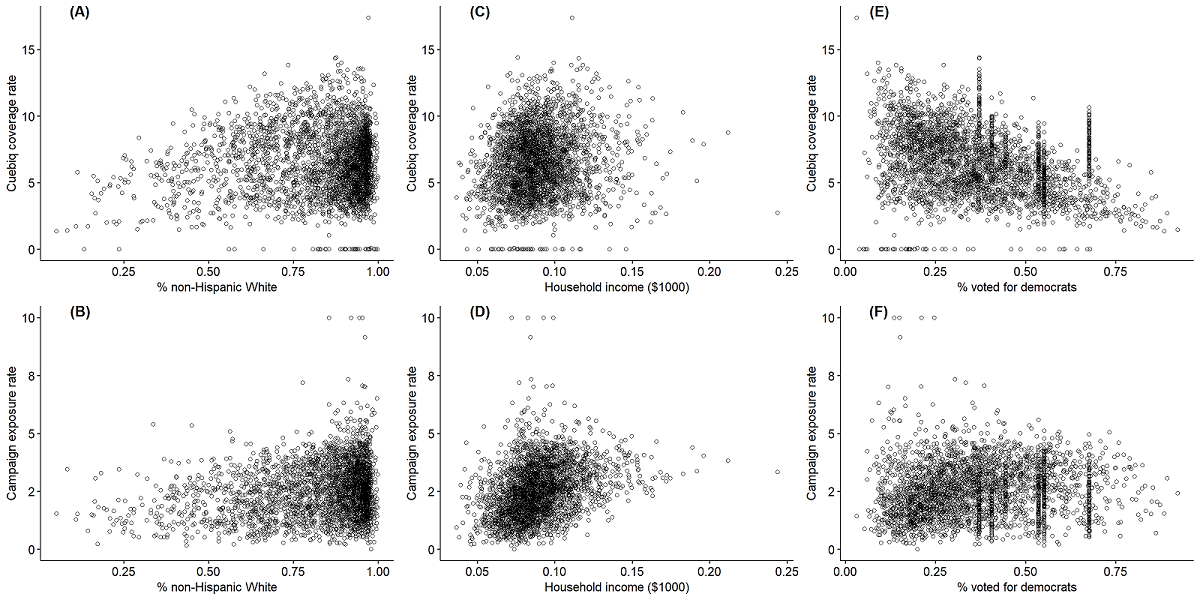
Cuebiq coverage and campaign exposure rate versus race, household income, and political affiliation during the first week of the study period. (A) Cuebiq coverage vs race; (B) Campaign exposure vs race; (C) Cuebiq coverage vs household income; (D) Campaign exposure vs household income; (E) Cuebiq coverage vs political affiliation; (F) Campaign exposure vs political affiliation.

Since this is not a randomized controlled trial, it is important to assess the potential selection issues in the data. Selection could have occurred in two stages. First, certain areas and groups may be overrepresented in the Cuebiq platform. This may be caused by different smartphone penetration rates by state and county. Second, the dissemination of Ad Council campaigns may be disproportionally concentrated in certain areas and groups. To assess the severity of selection, we plotted Cuebiq coverage rate and campaign exposure rate against race, income, and political affiliation at the county level (Figure 2). The Cuebiq coverage is higher in counties with a larger percentage of non-Hispanic White population. Campaign exposure rate has a similar correlation with race. A similar correlation is observed for household income. Richer counties tend to be overrepresented in the Cuebiq platform and get more exposure to the campaigns than poorer counties. For political affiliation, more democratic counties appear to be less represented in the Cuebiq platform, and there is not a strong pattern between campaign exposure and political affiliation. In sum, there may be potential selection issues in both Cuebiq platform coverage and campaign exposure. We will minimize the bias through accounting for these factors in the regression model.

The overall proportion of Cuebiq-enrolled devices exposed to the Ad Council campaigns started at 3.17% in the first week of the study period and gradually declined to 1.28% by the end of August 2021. A large number of channels were used to disseminate the campaign messages. Top channels in terms of exposure include The Trade Desk, Q Digital, Buzzfeed, Pandora, and Red Ventures.

**Figure 3:**
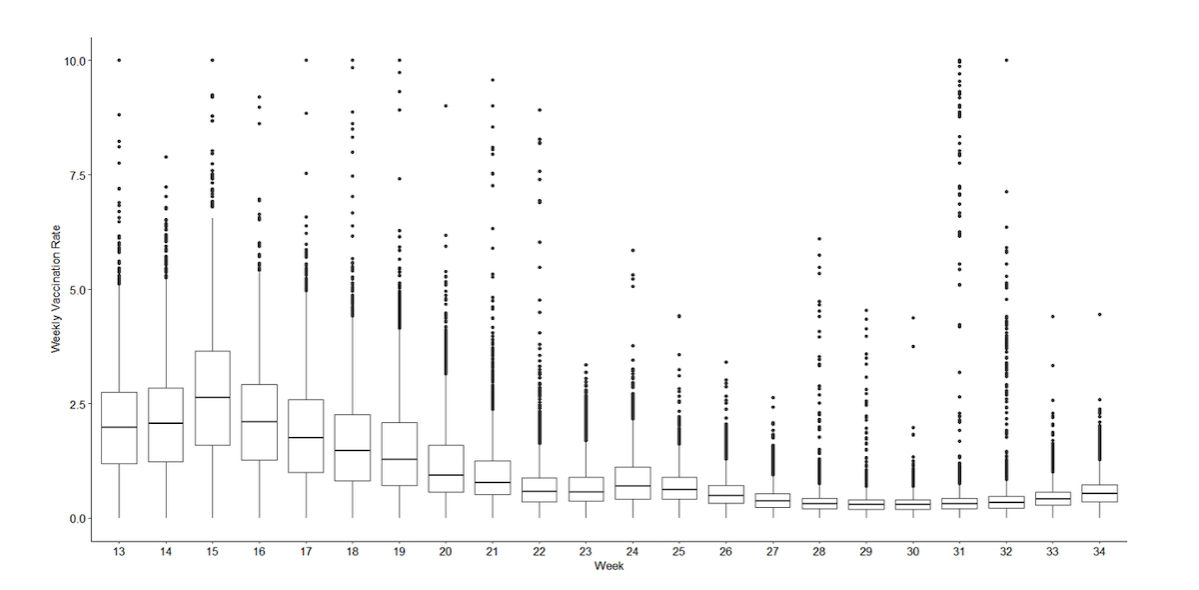
Distribution of county-level Centers for Disease Control and Prevention–reported weekly vaccination rate by week.

During the study period, the national vaccination rate increased from 16.4% to 47.2%, but the progress began slowing down (Figure 3) [23]. Overall, the weekly vaccination rate peaked in week 15 when 3.34% of Americans completed full vaccination that week; it dropped below 1% in later weeks. The box plots in Figure 3 illustrate the substantial heterogeneity in weekly vaccination rates across counties.

**Figure 4:**
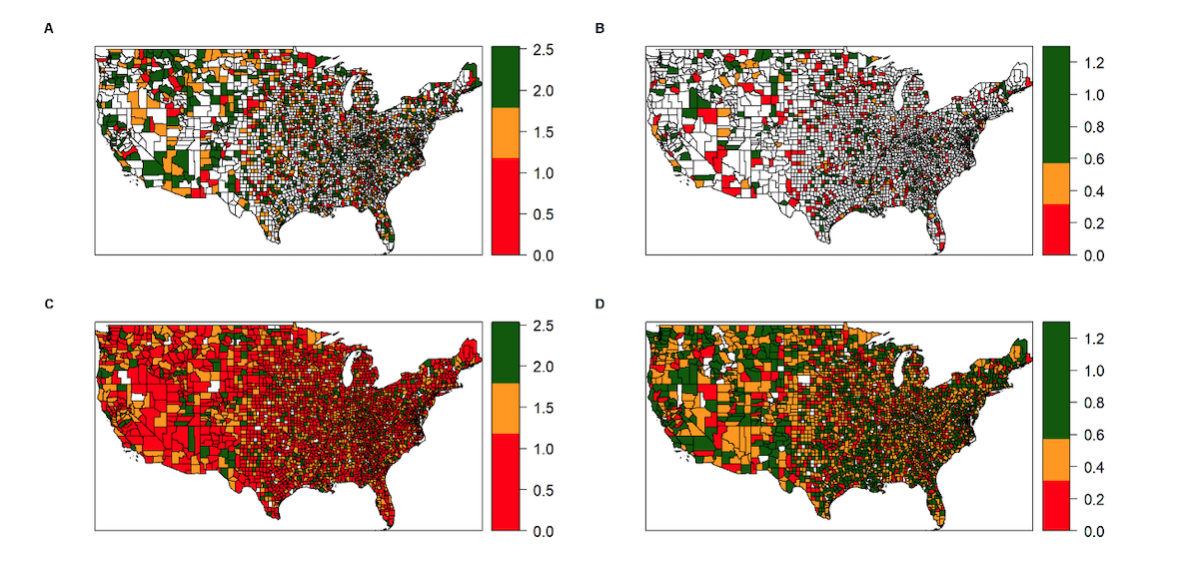
Exposure to the campaign and weekly vaccination rate by county during the first and last weeks of the study period. (A) Exposure rate (number of devices exposed to the campaign or total number of devices) to the campaign during the first week of the study period (week 13 or March 29 to April 4, 2021); (B) weekly vaccination rate during the first week of the study period; (C) exposure rate to the campaign during the last week of the study period (week 34 or August 12-29, 2021); (D) weekly vaccination rate during the last week of the study period.

Temporal autocorrelation in weekly vaccination rate is evident. Figure 4 illustrates the strong geographic clustering or spatial correlation in weekly vaccination rates. That spatial correlation may not fully be explained by the 3 variables in the model.

All indicators included in the Bayesian hierarchical model vary greatly across the 3104 counties analyzed in the study. The average percentage of the population aged 85 years and older is 2.3% (SD 0.01%) with a minimum of 0.0% and a maximum of 8.0% (Table 1). The ranges of the percentage of non-Hispanic White population and the percentage who voted for democrats are much wider at 5.1%-99.8% and 3.1%-82.1%, respectively. The poorest county earns an average of 36.5 thousand US dollars per household, while the richest county earns 243.8 thousand.

Table 2 presents our main results from the Bayesian hierarchical model with a spatiotemporally correlated residual structure. The coefficient for the exposure rate is 2.2 (95% uncertainty interval: 2.0-2.4). The effectiveness is impressive; exposing everyone in the county to the campaign may boost the vaccination uptake by 2.2% on a weekly basis. This effectiveness size is substantial, given the slow weekly vaccination rate of less than 1% at the end of the study period. It is worth noting that the effectiveness was assessed based on observed data. The exact magnitude of the effectiveness will likely change when the overall vaccination rates grow substantially higher in the future.

All factors in the model also have statistically significant impacts on vaccine uptake. Proportions of older population (≥85 years), non-Hispanic White population, democratic affiliation, and household income are all positively associated with vaccine rollout. This is consistent with findings from previous studies.

**Table 1:** Summary statistics of control variables for the 3104 counties in the study.

Variable	Mean (SD)	Min	Max
Population aged ≥85 years (%)	2.3 (0.01)	0.0	8.0
Non-Hispanic White (%)	83.3 (0.16)	5.1	99.8
Voted for democrats (%)	36.9 (0.17)	3.1	82.1
Household income (US $1000)	89.2 (20.0)	36.5	243.8

**Table 2:** Results from the Bayesian hierarchical model. Weekly data collected from 3104 counties during March 29 to August 29, 2021 (22 weeks); 95% uncertainty intervals were calculated from the posterior distributions.

Variable	Mean	95% uncertainty interval
Exposed to the campaign (%)	0.022	0.02 to 0.024
Population aged ≥85 years (%)	0.031	0.021 to 0.042
Non-Hispanic White (%)	0.004	0.003 to 0.004
Voted for democrats (%)	0.014	0.013 to 0.014
Median household income (US $1000)	0.058	0.054 to 0.062
Intercept	–0.005	–0.005 to –0.004

## Discussion

The study is one of the first rigorous evaluations of the “It’s Up to You” campaign, the largest effort in promoting COVID-19 vaccine awareness and uptake in the United States. We used Cuebiq’s nationwide location intelligence platform to monitor campaign exposure and then estimated its association with vaccine uptake data tracked by US Centers for Disease Control and Prevention. Results from the Bayesian hierarchical model support the effectiveness of the campaign in promoting vaccine uptake.

Under Operation Warp Speed, the COVID-19 vaccine research and development achieved great success at unprecedented scale and speed, thanks to the coordinated effort from governments, pharmaceutical companies, and other stakeholders [24]. The US Food and Drug Administration granted Emergency Use Authorization to 2 highly effective COVID-19 vaccines in late 2020. Since then, barriers to achieving herd immunity have shifted from medical to social.

On May 25, 2021, the United States marked the milestone of inoculating half of the adults in the country. Vaccinating the remaining population will be harder. Even before the milestone, vaccinations had been plateauing, and the official data had been chronicling a decreasing daily vaccination rate. Research and expert consensus suggest that as high as 80%-90% of the total population needs to be vaccinated to achieve herd immunity after considering recent factors, such as more contagious variants [25]. Given the high prevalence of vaccine hesitancy and uneven distribution across regions and populations, convincing the hesitant groups to get inoculated is necessary.

Overall, the already vaccinated population is better informed, less vaccine hesitant and skeptical, and more eager to be vaccinated than the remaining unvaccinated group. As the country approaches the vaccination rate required for herd immunity, vaccine resistance is also becoming more prominent in the remaining unvaccinated population. Reaching herd immunity requires vaccinating a significant proportion of the resistant population in addition to vaccinating all nonresistant populations. Thus, effective messaging campaigns are urgently needed. The root causes of vaccine hesitancy, such as fear, mistrust, and misconceptions, need to be addressed through targeted communication strategies.

The study has several limitations. First, the campaign was not designed to be a randomized control trial. People (or devices) were not randomly chosen to be exposed to the campaign messages. However, it is unlikely that there were systematic self-selection biases, given the passive nature of campaign exposure. Second, vaccine uptake requires a complex decision-making process that involves a potentially large number of factors. Due to limited data availability, our model may have missed some important variables that affect vaccine uptake. Third, as a county-level ecological analysis, it is challenging to make a causal inference. Lastly, the Cuebiq platform only monitors exposure to digital campaigns. Exposure to traditional media (eg, print publication, billboards, and posters) is not captured in the study.

Despite the limitations, the study is among the first efforts to evaluate the nation’s largest public health campaign. We built a fully validated model and established a statistically significant association between campaign exposure and vaccine uptake at the county level. The sizable effectiveness suggests that Ad Council is a sound investment of public and private resources. Given the low exposure and slow vaccination rate as of the end of the study period, expanding the messaging campaign may help accelerate vaccination progress.

The United States is no outlier when it comes to vaccine hesitancy, which was reported for nearly every vaccine and named one of the top ten threats to global health by the World Health Organization [26]. Misinformation about vaccines and antivax messages are global concerns. For example, a factor for Israel’s successful vaccination rollout is its multiprolonged educational campaigns that provide information, allay fears, and overcome hesitancy [27]. The successful implementation of the “It is Up to You” campaign and the documented effects in promoting vaccine uptake may generate valuable experience for other countries facing similar challenges.

## Abbreviations

CAR: conditional autoregressive

## Data Availability

Cuebiq data are available upon request from the Cuebiq Data for Good Program. All other data used in the study are publicly available from respective sources.
